# Effects of Using Immersive Media on the Effectiveness of Training to Prevent Ergonomics Risks

**DOI:** 10.3390/ijerph17072592

**Published:** 2020-04-10

**Authors:** Jose Antonio Diego-Mas, Jorge Alcaide-Marzal, Rocio Poveda-Bautista

**Affiliations:** 1 Instituto de Investigación e Innovación en Bioingeniería (I3B), Universitat Politècnica de València, 46022 València, Spain; jalcaide@dpi.upv.es; 2 Institute of Innovation and Knowledge Management (INGENIO) (CSIC-UPV), Universitat Politècnica de València, 46022 València, Spain; ropobau@dpi.upv.es

**Keywords:** ergonomics training, virtual reality, risk perception

## Abstract

In this work, the effects of using immersive media such as virtual reality on the performance of training programs to avoid ergonomics risks are analyzed. The advance of technology has made it possible to use low-cost portable devices able to generate highly immersive experiences in training programs. The effects of using this kind of device in training programs have been studied in several fields such as industrial security, medicine and surgery, rehabilitation, or construction. However, there is very little research on the effects of using immersive media in training workers to avoid ergonomics risk factors. In this study, we compare the effects of using traditional and immersive media in a training program to avoid three common ergonomics risk factors in industrial environments. Our results showed that using immersive media increases the participant’s engagement during the training. In the same way, the learning contents are perceived as more interesting and useful and are better remembered over time, leading to an increased perception of the ergonomics risks among workers. However, we found that little training was finally transferred to the workplace three months after the training session.

## 1. Introduction

Work related injuries, illnesses, and deaths are critical public health problems that result in important social and economic costs. In the European Union, the cost related to these problems is 3.3% of its gross domestic product (GPD), roughly reaching up to 3.9% of the worldwide GDP [1]. Musculoskeletal disorders (MSDs) are the most frequent problems and the leading cause of work disability, sickness, and absence from work [2,3]. For example, the prevalence of work related MSDs in Great Britain in 2017/18 was 469,000 out of a total of 1,358,000 for all work-related illnesses (35% of the total) [4]. It is difficult to measure the real economic burden related to MSDs [2,5]. Apart from direct costs, such as medical expenses or compensation, and indirect costs related to absenteeism or productivity decline, intangible costs due to the social consequences of MSDs must be considered [6]. Sixty-seven percent of those affected state that their quality of life has been significantly reduced [7] due to chronic pain, chronic fatigue, and economic reasons such as decreased incomes. Forty-nine percent of them declare that they are unable to perform their work normally, and 30% of them are worried about discrimination or losing their job [8].

Governments and public organizations encourage enterprises to adopt strategies to diminish the prevalence of MSDs. Physical, organizational, and cognitive ergonomics interventions are focused on reducing the risk factors for MSDs [9] through physical workplace redesigns, variations of task times and contents, implementing training programs, improving cognitive processes to reduce mental workload, and involving workers in developing and implementing changes [10]. Out of these interventions, currently, public occupational safety and health agencies and institutions are especially interested in promoting participatory ergonomic training programs. Ergonomic training programs are intended to transfer knowledge-relating ergonomics issues about work to the employees, but also to involve them in participating in the recognition and resolution of the problems [11].

There is very little research about the effectiveness of ergonomics training. In an effective ergonomics training program, the acquired knowledge must lead to a positive change in the trained workers’ behavior. The training is transferred to the workplace, achieving the goal of the training program, for example, a reduction of MSDs. However, there is some controversy about this. Some previous studies [12,13,14,15,16] support the effectiveness of occupational health and safety training. Educational interventions increase the safety knowledge of the workers, have a positive effect on attitude and beliefs toward prevention behaviors, and reduce negative safety and health outcomes like musculoskeletal pains and symptoms. On the other hand, some works and systematic reviews found that ergonomics training does not always lead to the expected workers’ behavioral change [17,18,19,20,21]. However, there is consensus that training methods and learning materials are important factors that affect the effectiveness of ergonomic training programs [14,22]. Findings from previous works revealed that as the method of training becomes more engaging, the effect of training is greater [12,13] and that the duration of outcome may be influenced by the style of training delivery. The perceived quality and usefulness of the training methods are positively correlated with learning transfer [23]. Furthermore, learning materials that are too theoretical prevent the transfer of training [24] and their effectiveness to change workers’ behavior is low [17,18]. Although moving from passive information-based methods (lectures, pamphlets, classroom theory lessons…) to more engaging methods results in greater knowledge acquisition and more transfer of training to the work setting, the most common training method in occupational health and safety is classroom theory lessons [14]. Currently, governments and public organizations promote the development of more engaging training methods and materials. Some examples are the series of occupational safety and health education toolkits (NAPO) devised by the European Agency for Safety and Health at Work, or projects like Train4work of the Federation of European Ergonomics Societies to identify training requirements and define learning objectives of the learning material in a participatory way. 

The development of information and communication technologies now makes new learning methods possible in training programs. Until recently, the use of these techniques implied the need to wear complex and expensive devices. However, the advance of technology has made it possible to use very low-cost portable devices able to generate highly immersive experiences. Some virtual reality (VR) headsets are currently available in the market for prices from 250 euros and are becoming common consumer products. Furthermore, smartphones can be used now as VR systems, using devices that transform any smartphone into a VR device for prices below 5 euros. On the other hand, developing content to be used in these devices is now cheap and affordable. Content generation systems have also evolved enormously in recent times and, nowadays, it is possible to develop immersive virtual environments (3D environments, 360° video, etc.) using free development frameworks.

Currently, due to these new low-cost VR devices and content development frameworks, learning methods that use technological tools and immersive media, such as VR or augmented reality, are extensively employed in training programs. The effects of using immersive media and contents on the performance of training programs have been studied in several fields such as industry [25,26,27], medicine and surgery [28,29,30], rehabilitation [31,32], or construction [33,34,35,36]. However, there is very little research on the effects of using immersive media in training workers to avoid ergonomics risk factors. In this work, we used traditional (video presentations) and immersive media (virtual reality headsets) in a training program to avoid three common ergonomics risk factors in industrial environments: repetitive movements, improper postures, and handling loads. The differences in using both ways of delivering the learning contents in the workers’ knowledge, behavior, and attitudes were analyzed. The results of this study may assist trainers in developing more effective training programs to avoid ergonomics risk factors.

## 2. Materials and Methods 

### 2.1. Participants and Settings

An automobile components supplier firm participated in this study through its Occupational Health and Safety Department (OHSD). The assembly plant of the enterprise develops its activity in two 8-h non-rotating shifts (hereafter called Sh1 and Sh2). There is no night shift and employees usually work in the same shift unless, unusually, there are special production requirements. Meeting the legislative framework, the workers of the assembly lines periodically receive training in health and safety matters. However, the training related to ergonomics was very basic and limited to less than one classroom hour.

The four assembly lines of the plant were analyzed and 36 workstations were selected. Several selected workstations were intended to perform the same production process and the tasks developed by the workers occupying them were the same. Thirty-three workstations were operated by one worker and three workstations needed two workers to run them. The 76 workers involved in the 36 workstations (38 workers from each shift) were contacted and invited to participate in the ergonomics training. Only the workstations whose workers from both shifts (Sh1 and Sh2) agreed to participate in the training were considered and, finally, 70 workers took part in the training. Each worker was randomly assigned to one out of two experimental groups (GrV and Gr3D) in such a way that the workers of the same workstation (in different shifts) were assigned to different groups. In this way, both experimental groups were composed of workers developing the same tasks in different shifts.

**Figure 1:**
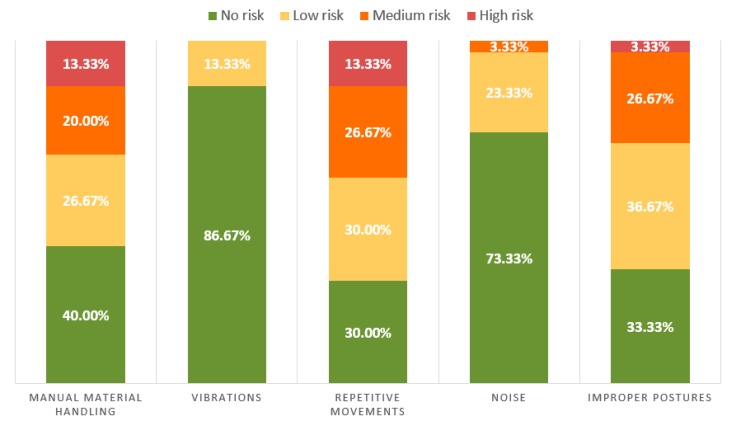
Percentage of workstations in each risk level for each ergonomics risk.

The OHSD staff of the enterprise were asked to classify each of the selected workstations in terms of several ergonomics risk factors (manual material handling, vibrations, repetitive movements, noise, and improper postures). The OHSD staff assigned a risk level (0—No risk, 1—Low risk, 2—Medium risk, and 3—High risk) to the tasks developed in each workstation for each ergonomics risk factor. The results of the classification showed that three risk factors were significant in the tasks developed by the workers participating in the study: manual material handling, repetitive movements, and improper postures (Figure 1).

### 2.2. Training Media and Contents

**Figure 2:**
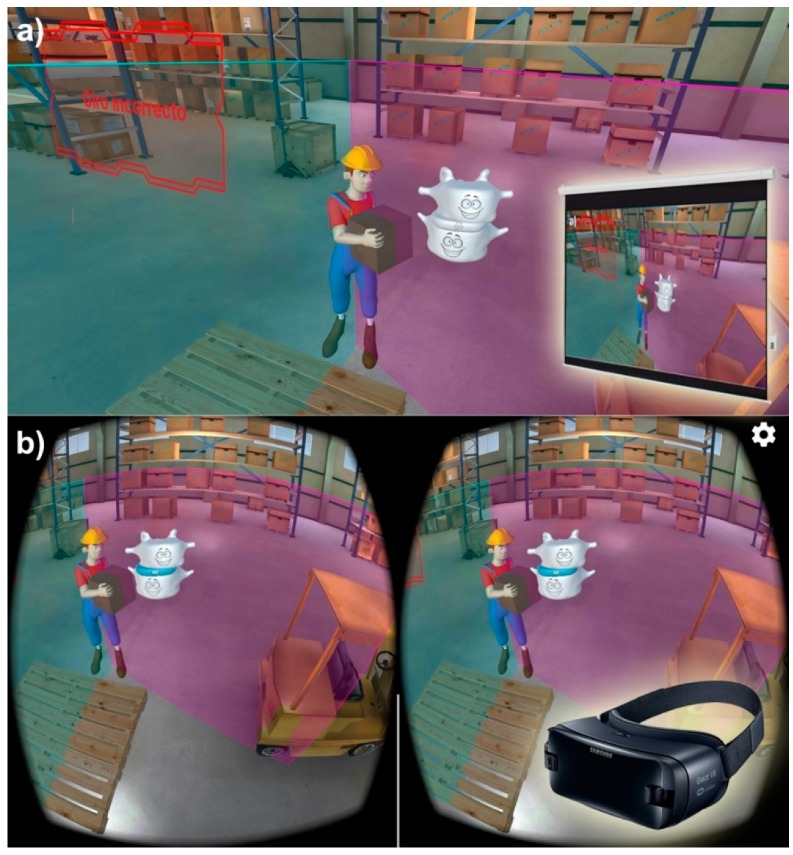
Media used to deliver the training: (**a**) Plain video presentation shown on a projection screen; (**b**) 3D interactive environment running on a virtual reality headset.

The most common ergonomics risk factors in European industrial environments are repetitive movements, improper postures, handling heavy loads, noise, and vibrations [37]. The last two factors were not significant among the workstations considered in this work (Figure 1). Therefore, we focused the contents of the training on the first three risk factors. Two different media were chosen to develop and deliver the training. The first one was a plain video presentation shown on a projection screen (Figure 2a), and the second was a 3D interactive environment running on a VR headset (Samsung Gear VR) (Figure 2b).

Unity, a cross-platform to create games and interactive experiences in 3D, was used to develop the training contents. Virtual industrial environments and a character used as an avatar representing the worker were created. The animated character was used to show the training contents for each risk factor (repetitive movements, improper postures, and handling heavy loads), developing different tasks in different work environments. For each risk factor, the character exposes ways to detect and identify the risk, their effects, and consequences on the workers’ health and personal life and good practices to avoid the risk.

### 2.3. Training Design and Evaluation

Seventy workers took part in the training. Two experimental groups (GrV and Gr3D) composed of 35 workers were created randomly, assigning each worker to one of the groups. The content of the training was presented to the group GrV using a plain video presentation shown on a projection screen. The workers of the group Gr3D used a VR headset (Samsung Gear VR). VR system users are prone to motion sickness when using a VR system. Motion sickness is a term used to describe the symptomatology of maladaptation syndrome, caused by various synthetic experiences, like using a VR system [38]. Therefore, three weeks before the training sessions, the workers of the group Gr3D were informed about the use of the VR headsets in the training and were asked if they had felt motion sickness if they used a VR system before. No worker declared ever having suffered motion sickness associated to using a VR system.

Kirkpatrick’s framework for training evaluation [39,40] was selected to measure the training effectiveness. Although there are more recent models to assess training programs [41,42,43], Kirkpatrick’s model is the most commonly used [44], acting as the fundamental scheme for educational evaluation [45,46]. Kirkpatrick’s framework proposes a model consisting of four stages. The first one (Reaction) measures the opinion of the participants about the training using questionnaires. The second (Learning) uses performance tests to measure the changes in the participants’ knowledge or skills. The third stage (Behavior) identifies if the new knowledge is being transferred to job behavior. Finally, the last step (Results) measures the organizational results and the cost and return on investment of the training. Three questionnaires and one knowledge test were designed in this work following Kirkpatrick’s model. The trainees answered the questionnaires, which will be described in the following paragraphs, using three different 7-point Likert-type scales: an agreement scale ranging from “strongly disagree” to “strongly agree”, a frequency scale ranging from “never” to “very frequently”, and an importance scale ranging from “not important at all” to “very important”. The scale used to answer each question is shown in the last column of Table 1, Table 2 and Table 3.

Each experimental group received the training in different sessions. All the workers of the GrV group received the training in one common session that lasted 2 h. Only 10 VR headsets were available, therefore the workers of the Gr3D experimental group were divided into three equal training sessions, each lasting 2 h. The sessions were conducted by members of the OHSD staff of the company. Initially, the trainers presented the objectives of the sessions and the participants filled out the PQ questionnaire (Table 1). This questionnaire consisted of 11 questions divided into 5 groups, hereafter named dimensions (Concern, Control, Loads, Repetitiveness, and Postures). The Concern dimension (questions PQ1 to PQ3) was intended to measure the level of the workers’ concern about ergonomics risks in their tasks, while the Control dimension (questions PQ4 and PQ5) assesses if the workers perceive that ergonomics risks in their workplaces are under control, because they can act on them or because managers of health and safety are dealing with them. The dimensions Loads, Repetitiveness, and Postures (questions PQ6 to PQ7, PQ8 to PQ9, and PQ10 to PQ11) are similar to the Concern dimension but measure the workers’ concern about each specific ergonomics risk selected for this study.

Then, participants of the GrV group watched the contents of the training in a video presentation shown on a projection screen. The workers of the Gr3D group watched the immersive and interactive version of the content. Previously, they were instructed on using the VR headset and the way to interact with the contents. After watching the contents, the participants and the trainers discussed ergonomics risks in their workplaces. Next, the workers filled out the RQ questionnaire (Table 2) related to the Reaction stage of Kirkpatrick’s model. This questionnaire consisted of 6 questions intended to measure the reaction of the participants about the received training. The first one, RQ1 (Had you ever used a VR headset before this training?) was only presented to the Gr3D group members and it was intended to measure the degree of novelty of this kind of device for the participants. A Likert-type scale was not used to answer this question. Three options were given: “No. I had never used a VR headset”, “Yes. I had used a VR headset only once” and “Yes. I had used a VR headset more than once”. The reaction of the participants was assessed in three dimensions: Expectation (RQ2), Interest (RQ3 and RQ4), and Usefulness (RQ5 and RQ6). The participants’ expectations about the training has a significant impact on post-training attitudes and motivation to transfer learning [47]. On the other hand, Interest dimension measures the level of engagement of the participants during the training sessions, which affects the effectiveness of ergonomics training programs [12,13,14,22]. Finally, the perceived usefulness of the training methods are positively correlated with learning transfer [23]. 

**Figure 3:**
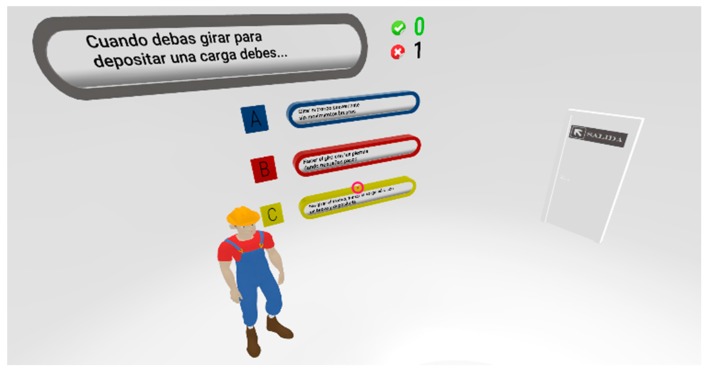
3D interactive multiple-choice test (LT) running on a VR headset.

The learning level of Kirkpatrick’s framework for training evaluation [39] offers data on the extent to which the knowledge or skills of the participants have changed through a knowledge test. In this work, a multiple-choice test (LT) consisting of 30 questions (10 about each ergonomics risk factor) with three alternatives was used. The participants answered the LT test twice. One of them was filled out immediately after the presentation of the learning contents and the second one was answered three months later. The first time, the participants of the Gr3D experimental group, who received the training using the VR headset, filled out the test in the same application used to view the learning contents in 3D (Figure 3). The members of the GrV group filled out a web form. In the second round, all the groups used the web-based version of the test.

**Figure 4:**
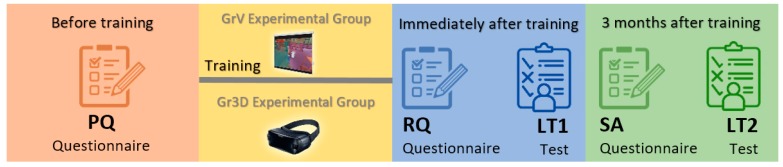
Sequence of the surveys.

Three months after the training, the participants filled out the SA questionnaire (Table 3). This was a self-assessment questionnaire intended to appraise the workers’ self-perceived behavioral changes after the training and the learning transferred to the work place. Kirkpatrick [39] recommends a minimum term of three months after the training for the behavioral appraisal, and Axtell et al. [48] showed that the amount of learning transferred one month or later after the training is a good predictor of the learning transferred after one year. The questions of the SA questionnaire were grouped in 5 dimensions: Memory, Concern, Control, Transference, and Usefulness. The Memory dimension (questions SA1 and SA2) measured if the trainees had remembered the learning contents after the training. The Concern (questions SA3 to SA5) and the Control (questions SA6 and SA7) dimensions were the same used in the PQ questionnaire. To differentiate the answers given before and after the training, in Table 1, the Concern and Control dimensions are named Concern PreT (pre-training) and Control PreT, respectively. In the same way, the Usefulness dimension (questions SA11 and SA12) was the same used in the RQ questionnaire. To differentiate the answers given just after the training and three months after the training, in Table 2, the Usefulness dimension is named Usefulness PosT (post-training), and in Table 3, it is named Usefulness 3M. Figure 4 shows the sequence in which the questionnaires were filled out.

## 3. Results

**Figure 5:**
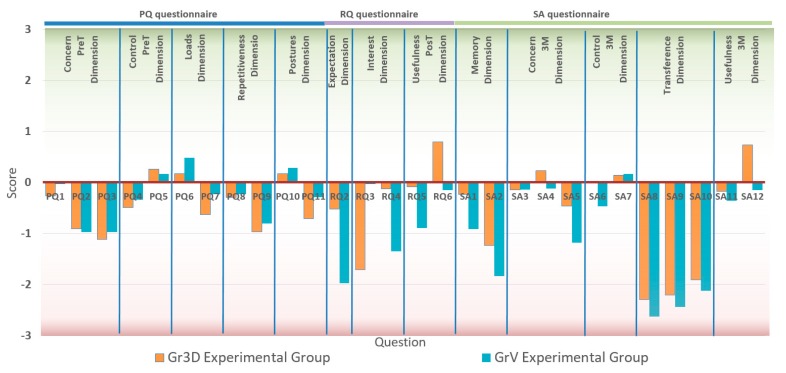
Average response to each question by experimental group.

Initially, 70 workers took part in the training and answered the PQ and RQ questionnaires and the LT knowledge test. One of the participants of the GrV group (GrV26 in Table A3) did not complete the LT test and the SA questionnaire in the second round because he no longer worked in the company. Figure 5 shows the average response to each question by experimental group. To elaborate this figure, a score was given to each answer of the scales ranging from −3 for the lowest level of the scales (strongly disagree, never, and not important at all) to +3 for the highest level (strongly agree, very frequently, and very important).

A different score system was used to statistically analyze the data. For the lowest level of the scales, 1 was used, and for the highest level of the scales, 7 was used. The complete set of responses to the questionnaires PQ and RQ are shown in Table A1 and Table A2 in the Appendix A using this score system. The results for the questionnaire SA and for the LT knowledge tests are given in Table A2 and Table A3. In these tables, the LT1 column shows the number of correct answers given to the LT test in the first round, and the LT2 column shows the difference between the number of correct answers given to the LT test in the first round and in the second round three months after the training. 

Table 4 shows the mean and the standard deviation of the responses to each question by experimental group, and the results of the *t*-tests conducted to check whether there are statistically significant differences between the two groups (at significance level of 0.05). The scores for each dimension (Concern, Control, Loads, Repetitiveness, Postures, Expectation, Interest, Usefulness, Memory, and Transference) were calculated, summing up the scores of the correspondents’ questions for each participant. Questions PQ5 and RQ3 were negatively worded with respect to the rest of the questions of their dimensions, therefore, the scores of these questions were reversed. Cronbach’s alpha (α) was used to calculate the internal reliability of the dimensions. The Cronbach’s alpha is commonly used to test for internal consistency and reliability of a questionnaire consisting of multiple Likert scales and items [49]. Although there is no clear consensus on the value of α that indicates an acceptable reliability, a value over 0.7 is usually considered to indicate a moderate consistency [50]. The Cronbach’s alphas in this study (Table 4) showed that all the dimensions had a moderate internal reliability.

The results of the PQ questionnaire (Table 4) showed that there were not significant differences between the workers of the two experimental groups before the training. No significant differences were found (*t* (68) = −0.37, *p* = 0.714) in the level of the workers’ concern about ergonomics risks in their tasks (Concern dimension) between the workers selected to receive the training using a video projection (GrV: M = 10.03) and the workers selected to receive the training using a VR headset (Gr3D: M = 9.71). Differences between the groups were not found in any of the questions of this dimension either. The scores of the questions PQ1 (GrV: M = 3.97, Gr3D: M = 3.74), PQ2 (GrV: M = 3.03, Gr3D: M = 3.09), and PQ3 (GrV: M = 3.03, Gr3D: M = 2.89) show, in general terms, a low workers’ level of concern about ergonomics risks and the consequences on their long-term health. In the same way, no differences were found between experimental groups in the Control dimension (*t* (68) = −0.40, *p* = 0.687) before the training. The answers to the questions PQ4 (GrV: M = 3.66, Gr3D: M = 3.51) and PQ5 (GrV: M = 3.83, Gr3D: M = 3.74) showed that workers believed that ergonomics risks were controlled to the same extent by the managers of health and safety of the plant and by themselves. Improperly handling loads (GrV: M = 4.48, Gr3D: M = 4.17) and adopting awkward postures (GrV: M = 4.29, Gr3D: M = 4.17) were considered more important risk factors than performing excessive repetitive movements (GrV: M = 3.77, Gr3D: M = 3.71), with no differences between the experimental groups. Nonetheless, the answers to the questions PQ7 (GrV: M = 3.77, Gr3D: M = 3.37), PQ9 (GrV: M = 3.20, Gr3D: M = 3.03), and PQ11 (GrV: M = 3.71, Gr3D: M = 3.19) showed that they did not feel that they needed training to avoid any of the risk factors.

Conversely, the answers to the RQ questionnaire, filled out just after the training, showed significant differences between the two experimental groups. From the results of the RQ2 question, the participants of the Gr3D group were significantly (*t* (68) = 5.03, *p* < 0.001) more keen on receiving the training than those of the GrV group (GrV: M = 2.03, Gr3D: M = 3.48). In the same way, significant differences (*t* (68) = 7.53, *p* < 0.001) between the experimental groups were found in the level of engagement of the participants during the training sessions (Interest dimension). The participants of the GrV group felt more tired or bored during the training (question RQ3) than those of the Gr3D group (GrV: M = 4.03, Gr3D: M = 5.71). Similarly, judging from the RQ4 question’s answers, the participants who used the VR headset found the learning materials more interesting (GrV: M = 2.66, Gr3D: M = 3.88). Finally, the perceived usefulness of the training (Usefulness PosT dimension) showed significant differences (*t* (68) = 5.40, *p* < 0.001) between the groups. The questions RQ5 and RQ6 indicate that the workers who used the VR headset found the training more useful (GrV: M = 3.11, Gr3D: M = 3.91) and better than previous similar trainings (GrV: M = 3.85, Gr3D: M = 4.80). The scores of the LT knowledge test (LT1 column in the Table A3 and Table A4) were a bit higher for the participants of the GrV experimental group (GrV: M = 23.37, Gr3D: M = 22.06), but this difference was not significant (*t* (68) = 1.68, *p* < 0.397).

Significant differences were found between the groups (*t* (67) = 2.24, *p* = 0.029) in the Memory dimension of the SA questionnaire, filled out three months after the training. The Memory dimension measured if the trainees remembered the learning contents after the training, when they were undertaking their tasks (SA1 question), and if they analyzed these tasks considering the learning of the training (SA2 question). The results showed that the workers who received the training using the VR headset remembered the training more than those in the GrV group (GrV: M = 5.26, Gr3D: M = 6.54).

The Concern dimension was assessed again in the SA questionnaire (Concern 3M in Table 4). Three months after the training, significant differences (*t* (67) = 2.04, *p* = 0.045) were found between groups in this dimension. The workers of the Gr3D group showed a higher level of concern about ergonomics risks and the consequences on their long-term health (GrV: M = 9.91, Gr3D: M = 11.69). An analysis of covariance (ANCOVA) was performed on the Concern 3M scores, using the scores of Concern PT as the covariate, to check if the media used to deliver the training significantly affected the concern of the workers about ergonomics risks. The ANCOVA indicated that the training method produced significant differences in the Concern dimension (Table 5) at the 0.05 level of probability.

Conversely, no significant differences between the groups were found in the Control dimension three month after the training (*t* (67) = 0.83, *p* = 0.411) and neither were they found in the scores of the questions in this dimension (SA6 and SA7). An ANCOVA performed on the Control 3M scores, with the scores of Control PT as the covariate, found that the media used to deliver the training did not affect the workers perception of the ergonomics risks in their workplaces (*F* (1, 66) = 2.25, *p* = 0.138).

The results of the Transference dimension showed that little training was transferred to the work place, and no significant differences were found between the experimental groups (*t* (67) = 1.76, *p* = 0.083). The workers of the Gr3D group were able to identify risky activities among their tasks after the training to a greater extent than the participants in the GrV group (GrV: M = 1.38, Gr3D: M = 1.71). However, from the scores of questions SA9 and SA10, there were no differences between the groups in how the participants tried to improve the way in which they undertook their tasks to avoid ergonomics risks.

As occurred immediately after the training, three months later the perceived usefulness of the training (Usefulness 3M) showed significant differences (*t* (67) = 2.36, *p* = 0.021) between the experimental groups. However, the differences were, in this case, limited to only one out of the two questions in this dimension. Although the participants in the Gr3D group found the training significantly better than similar ones received previously (*t* (67) = 3.81, *p* < 0.001), no differences between the groups were found in the perceived usefulness of the training (*t* (67) = 0.60, *p* = 0.549).

**Figure 6:**
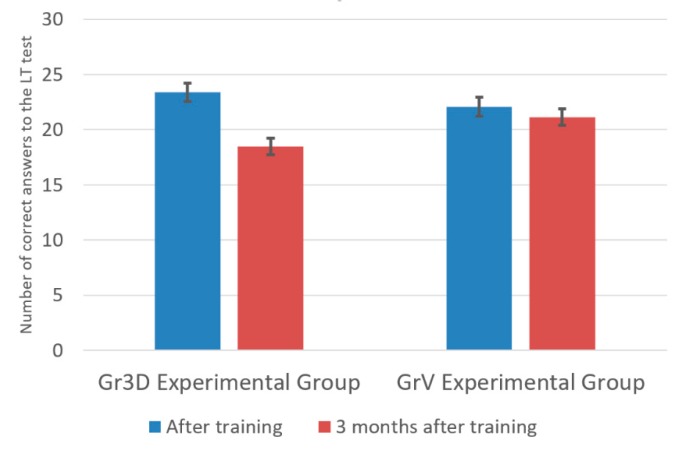
Number of correct answers (and standard error) given to the LT test just after the training and three months after the training.

As aforementioned, there were no significant differences between the groups in the scores of the LT knowledge test filled out just after the training. Therefore, it seems that the media used to deliver the training did not affect the level of knowledge or skills acquired by the participants. However, the results of the same test filled out three months after the training showed significant differences between the groups (*t* (67) = 3.11, *p* = 0.003). The LT2 column in Table A3 and Table A4 showed the difference between the number of correct answers given to the LT test in the first round and in the second round. Although the results were similar for the participants of the two groups in the first round, the scores in the second round (GrV: M = 18.47, Gr3D: M = 21.14) were better for the participants who used the VR headset during the training (Figure 6). An ANCOVA was performed on the LT1 scores with the scores of LT2 as the covariate. The results (*F* (1, 66) = 9.79, *p* = 0.003) showed that the media used to deliver the training significantly affects how the knowledge acquired was retained over time.

## 4. Discussion

In this work, we studied the effects of using immersive media to deliver the learning contents in ergonomics training on the workers’ knowledge, behavior, and attitudes about ergonomics risk factors. The differences in using a traditional video projector and a VR headset were analyzed. The data were collected immediately after the training and three months later.

To achieve the training transfer to the workplace, the worker must be aware of the consequences of ergonomics risks on their health and quality of life. An adequate perception of the ergonomics risks is needed. The perceived risk is the subjective judgment that the worker makes about the frequency of a particular risk and the severity of the consequences [51]. In general terms, the level of concern about ergonomics risks and the consequences on the long-term health of the workers who participated in the study was low. They did not feel that they needed training to avoid any of the risk factors selected for this study despite there being risks present in their tasks (more than 30% of the workstations had a medium or high level of each kind of risk). The results of this study found that the media used to deliver the training significantly affects the change in the workers’ level of concern about ergonomics risks. The workers of the experimental group that used a VR headset showed a higher level of concern about ergonomics risks and their consequences on their long-term health three months after the training. A low level of concern about ergonomics risks is a common situation because, overall, work-related musculoskeletal disorders are cumulative traumas. The injuries are caused by the accumulation of small traumas over a long period of time. Therefore, it is difficult for the worker to establish a direct cause–effect relationship between the ergonomics risk factors and their consequences for their health. Using immersive media in ergonomics training may lead to an increased ergonomics risks perception.

Although the workers of both experimental groups did not feel they needed the training, we found significant differences in the participants’ expectations about the training due to the different media used to deliver the learning contents (the only difference between the two experimental groups). The workers assigned to the training group that used the VR headset were significantly keener on receiving the training than those of the GrV group. This could have a significant influence on post-training attitudes and motivation to transfer learning [47]. In the same way, significant differences in the level of engagement of the participants during the training sessions were found. During the training, the participants who used the VR headset found the learning materials more interesting, while the participants of the GrV group felt more tired or bored. The degree of novelty of the technology used in the training can affect the engagement of the trainees [52], and the level of engagement of the participants during the training sessions affects the effectiveness of training programs [12,13,14,22]. Therefore, part of the effectiveness of the training can be attributed to the novelty of the device [53]. Our intention was to measure if the different degrees of novelty of the VR headset among the workers of the Gr3D experimental group affected the effectiveness of the training. However, the answers to the question RQ1 of the RQ questionnaire (Table 4) show that 88.57% of the workers of the Gr3D experimental group had never used a VR headset before the training, only 3 workers had used it once, and only 1 more than once. Therefore, there were not enough participants with experience in the use of VR headsets to perform a significant statistical analysis on the influence of novelty on training effectiveness.

Just after the training, the workers who used the VR headset found the training more useful and better than previous similar training. The perceived quality and usefulness of the training methods are positively correlated with learning transfer [23]. Although three months later the differences were limited to training quality and no differences between the groups were found in the perceived usefulness of the training, overall, the scores of the Usefulness dimension were significantly higher in the Gr3D experimental group. On the other hand, although the media used in the training does not seem to have an effect on the level of knowledge or skills acquired by the participants, the learning was better retained over time by the workers who used the VR headset. This result is consistent with the findings of previous works that revealed that the duration of the outcome of the training may be influenced by the style of training delivery, lasting longer as the method of training becomes more engaging [12,13].

Despite the results in the other analyzed dimensions, the Transference dimension shows no significant differences between the experimental groups. Although the workers who used the VR headset were better able to identify risky activities among their tasks, they did not try to improve the way in which they undertook their tasks to avoid the perceived ergonomics risks. Therefore, from the responses of the participants of both groups, little training was transferred to the work place. 

The low level of concern of the workers about ergonomics risks and their consequences may be a key aspect in the low level of transference found. The realization of the severity of the risks is essential so that the workers be motivated to learn about the risks and to transfer the knowledge to the work setting [54] Using immersive media in the training program has been found to increase the workers’ risk perception to a greater extent than traditional procedures. However, the overall level of concern of the workers about ergonomics risks remains low after the training. 

Finally, a limitation of this work must be pointed out. The participants in the study were assigned to each experimental group in a quasi-randomized way. Each participant was randomly assigned to one out of two experimental groups (GrV and Gr3D) in such a way that the workers of the same workstation (in different shifts) were assigned to different groups. In this way, both experimental groups were composed of workers developing the same tasks in different shifts. However, potential differences between the experimental groups can be of importance for the results obtained in this work.

## 5. Conclusions

Our results showed that using an immersive media in ergonomics training increases the workers’ expectations about the training session and the participant’s engagement. In the same way, learning contents are perceived as more interesting and useful and are better remembered over time. Using immersive media may lead to an increased workers’ ergonomics risks perception, however, we found that little training is finally transferred to the workplace three months after the training session. From a practical perspective, using low-cost immersive media in ergonomics training produces significant benefits. However, this training modality does not increase the workers’ risk perception enough to significantly improve the knowledge transferred to the work setting. Therefore, other measures must be taken so that the workers establish a direct cause–effect relationship between ergonomics risk factors and their severe consequences for their health.

## Figures and Tables

**Table 1:** Questions of the PQ questionnaire.

Dimension	Question Code	Question	Scale
Concern PreT	PQ1	Your tasks could have consequences for your long-term health because of ergonomics risks.	Agreement
	PQ2	How often did you feel worried about the consequences of some ergonomics bad practices on your long-term health?	Frequency
	PQ3	The importance of the harm that ergonomics risks can cause you is...	Importance
Control PreT	PQ4	If some aspects of your task must be changed to avoid ergonomics risk they are out of your control.	Agreement
	PQ5	Managers of health and safety in your company know and control the risks associated with ergonomics.	Agreement
Loads	PQ6	The importance of the consequences of handling loads improperly for your health is…	Importance
	PQ7	You need training on handling loads correctly.	Agreement
Repetitiveness	PQ8	The importance of the consequences of performing excessive repetitive movements for your health is…	Importance
	PQ9	You need training on avoiding the effects of repetitive movements.	Agreement
Postures	PQ10	The importance of the consequences of adopting bad postures during your working hours for your health is…	Importance
	PQ11	You need training on maintaining good posture in your task.	Agreement

**Table 2:** Questions of the RQ questionnaire.

Dimension	Question Code	Question	Scale
-	RQ1	Had you ever used a VR headset before this training?	
Expectation	RQ2	You were keen on receiving the training.	Agreement
Interest	RQ3	You felt tired or bored during training.	Agreement
	RQ4	The learning materials are interesting.	Agreement
Usefulness PosT	RQ5	This training is useful for your work activity.	Agreement
	RQ6	This training is better than previous ones on the same issue you have received.	Agreement

**Table 3:** Questions of the SA questionnaire.

Dimension	Question Code	Question	Scale
Memory	SA1	You have remembered the ergonomics training received three months ago when undertaking your tasks.	Frequency
	SA2	You have analyzed your task considering the learning of the training.	Agreement
Concern 3M	SA3	Your tasks could have consequences for your long-term health because of ergonomics risks.	Agreement
	SA4	How often did you feel worried about the consequences of some ergonomics bad practices on your long-term health?	Frequency
	SA5	The importance of the harm that ergonomics risks can cause you is...	Importance
Control 3M	SA6	If some aspects of your task must be changed to avoid ergonomics risk they are out of your control.	Agreement
	SA7	Managers of health and safety in your company know and control the risks associated with ergonomics.	Agreement
Transference	SA8	You have identified some risky activity among your tasks that you had not realized before the ergonomics training.	Agreement
	SA9	You have changed some aspect of the way you undertake your task to avoid ergonomics risks after the training.	Agreement
	SA10	The way you undertake your tasks after the training is better for your health than before.	Agreement
Usefulness 3M	SA11	This training is useful for your work activity.	Agreement
	SA12	This training is better than previous ones on the same issue you have received.	Agreement

**Table 4:** Mean and standard deviation of the responses to each question by experimental group, and Cronbach’s alphas of the questions of each dimension (significance level of 0.05).

Dimension	Question	Cronbach’s Alpha	GrV Group Scores Mean (SD)	Gr3D Group Scores Mean (SD)	*t*	*p*-Value
Concern PreT		0.92	10.03 (3.75)	9.71 (3.38)	−0.37	0.714
	PQ1	-	3.97 (1.29)	3.74 (1.17)	−0.77	0.441
	PQ2	-	3.03 (1.25)	3.09 (1.17)	0.20	0.844
	PQ3	-	3.03 (1.42)	2.89 (1.37)	−0.43	0.670
Control PreT		0.71	7.49 (2.25)	7.26 (2.47)	−0.40	0.687
	PQ4	-	3.66 (1.51)	3.51 (1.36)	−0.42	0.679
	PQ5 *	-	3.83 (1.10)	3.74 (1.38)	−0.29	0.774
Loads		0.85	8.26 (1.93)	7.54 (2.31)	−1.41	0.164
	PQ6	-	4.48 (1.07)	4.17 (1.18)	−1.17	0.250
	PQ7	-	3.77 (1.06)	3.37 (1.26)	−1.44	0.156
Repetitiveness		0.73	6.97 (2.19)	6.74 (2.17)	−0.44	0.660
	PQ8	-	3.77 (1.33)	3.71 (1.23)	−0.19	0.852
	PQ9	-	3.20 (1.18)	3.03 (1.17)	−0.61	0.545
Postures		0.83	8.00 (2.09)	7.46 (2.31)	−1.03	0.305
	PQ10	-	4.29 (1.18)	4.17 (1.17)	−0.41	0.686
	PQ11	-	3.71 (1.13)	3.29 (1.27)	−1.49	0.141
Expectation	RQ2	-	2.03 (0.86)	3.48 (1.48)	5.03	<0.001 **
Interest		0.73	6.69 (1.37)	9.60 (1.83)	7.53	<0.001 **
	RQ3 *	-	4.03 (0.89)	5.71 (0.96)	7.63	<0.001 **
	RQ4	-	2.66 (0.97)	3.88 (1.16)	4.82	<0.001 **
Usefulness PosT		0.70	6.97 (0.98)	8.71 (1.64)	5.40	<0.001 **
	RQ5	-	3.11 (0.63)	3.91 (1.07)	3.82	<0.001 **
	RQ6	-	3.85 (0.65)	4.80 (0.80)	5.43	<0.001 **
Memory		0.86	5.26 (2.13)	6.54 (2.58)	2.24	0.029 **
	SA1	-	3.09 (1.36)	3.77 (1.39)	2.06	0.431
	SA2	-	2.17 (1.03)	2.77 (1,26)	2.14	0.036 **
Concern 3M		0.89	9.91(3.49)	11.69 (3.71)	2.04	0.045 **
	SA3	-	3.20 (1.27)	3.86 (1,40)	2.02	0.047 **
	SA4	-	3.88 (1.41)	4.29 (1,18)	1.29	0.201
	SA5	-	2.82 (1.24)	3.54 (1.46)	2.20	0.031 **
Control 3M		0.72	7.71 (2.49)	8.14 (1.87)	0.83	0.411
	SA6	-	3.53 (1.21)	4.00 (1.00)	1.76	0.083
	SA7	-	4.17 (1.60)	4.14 (1.06)	−0.10	0.918
Transference		0.76	4.82 (1.71)	5.60 (1.94)	1.76	0.083
	SA8	-	1.38 (0.60)	1.71 (0.71)	2.09	0.041 **
	SA9	-	1.56 (0.66)	1.80 (0.68)	1.50	0.139
	SA10	-	1.88 (0.88)	2.09 (0.95)	0.92	0.360
Usefulness 3M		0.71	7.50 (1.38)	8.57 (2.28)	2.36	0.021 **
	SA11	-	3.64 (1.01)	3.83 (1.44)	0.60	0.549
	SA12	-	3.85 (0.78)	4.74 (1.12)	3.81	<0.001 **

* Inverted scores, ** Correlation statistically significant at *p* < 0.05.

**Table 5:** Analysis of covariance for means comparing the scores of the Concern dimension three month after the training based on training methods.

Source of Variance	Degrees of Freedom (d*f*)	Mean Sum of Squares (MS)	*F*	*p*-Value
**Concern PreT**	1	662.24	210.10	<0.001
**Training method**	1	77.89	24.71	<0.001
**Residuals**	66	3.15		
**Adjusted total**	68	-		

## Appendix A

**Table A1:** Responses to the PQ and RQ questionnaires given by the GrV experimental group.

Questionnaire	PQ											RQ					
Question	PQ1	PQ2	PQ3	PQ4	PQ5	PQ6	PQ7	PQ8	PQ9	PQ10	PQ11	RQ1	RQ2	RQ3	RQ4	RQ5	RQ6
**Participant**																	
**GrV1**	4	3	4	4	5	4	4	5	3	3	4	-	2	3	5	3	3
**GrV2**	5	4	3	4	4	4	3	4	2	3	3	-	4	3	5	3	3
**GrV3**	4	3	4	3	5	4	4	2	3	4	4	-	3	4	4	4	3
**GrV4**	3	2	1	4	3	4	3	3	2	4	3	-	2	4	4	1	3
**GrV5**	4	3	2	3	5	5	4	3	4	5	5	-	2	5	3	2	2
**GrV6**	3	2	3	3	3	4	4	4	4	4	4	-	2	4	4	2	2
**GrV7**	2	1	1	1	5	3	2	2	1	3	2	-	3	5	3	4	4
**GrV8**	5	4	4	4	3	4	5	6	4	4	5	-	2	3	5	3	3
**GrV9**	2	1	2	2	4	2	4	1	3	1	3	-	2	3	5	3	3
**GrV10**	5	4	5	5	4	6	5	5	4	6	5	-	2	4	4	3	3
**GrV11**	4	3	2	4	5	4	4	5	4	4	4	-	3	6	2	4	4
**GrV12**	5	4	3	6	2	6	4	6	3	6	4	-	4	3	5	5	4
**GrV13**	3	2	3	3	6	5	4	3	4	5	4	-	1	4	4	2	3
**GrV14**	5	4	4	5	3	6	5	5	5	5	5	-	2	5	3	3	3
**GrV15**	5	4	2	4	5	4	3	3	3	3	3	-	1	3	5	2	2
**GrV16**	4	3	3	3	5	5	6	4	5	5	6	-	2	4	4	2	3
**GrV17**	4	3	4	4	5	5	4	3	3	5	3	-	2	5	3	1	3
**GrV18**	6	5	5	6	4	6	5	5	5	6	5	-	2	5	3	2	2
**GrV19**	1	1	1	1	5	3	1	3	1	3	1	-	3	4	4	3	3
**GrV20**	3	2	2	1	4	3	2	3	2	3	2	-	2	3	5	3	3
**GrV21**	6	5	6	6	3	5	3	5	2	5	3	-	3	6	2	2	3
**GrV22**	4	3	4	3	6	5	4	3	3	5	4	-	2	4	4	4	4
**GrV23**	5	4	3	5	3	6	5	6	5	6	5	-	2	5	3	4	4
**GrV24**	4	3	3	3	5	5	4	4	4	5	4	-	3	4	4	3	4
**GrV25**	4	3	4	4	6	4	4	3	3	4	4	-	1	3	5	1	3
**GrV26**	3	2	2	3	5	4	2	4	2	3	2	-	1	3	5	3	4
**GrV27**	7	6	7	7	3	6	5	5	5	6	5	-	2	4	4	2	3
**GrV28**	3	2	2	3	4	5	4	4	4	5	4	-	1	4	4	1	3
**GrV29**	4	3	4	4	5	4	4	3	3	4	4	-	1	4	4	3	3
**GrV30**	4	4	2	4	3	5	3	5	2	5	3	-	2	3	5	3	3
**GrV31**	3	2	1	4	2	4	3	3	2	4	2	-	1	4	4	2	3
**GrV32**	6	5	4	6	3	6	5	5	5	5	5	-	1	4	4	2	2
**GrV33**	2	1	2	2	5	3	3	2	2	3	3	-	3	3	5	3	4
**GrV34**	3	2	2	1	4	3	3	1	2	3	3	-	1	5	3	2	3
**GrV35**	4	3	2	3	4	5	4	4	3	5	4	-	1	3	5	3	4

**Table A2:** Responses to the PQ and RQ questionnaires given by the Gr3D experimental group.

Questionnaire	PQ										RQ						
Question	PQ1	PQ2	PQ3	PQ4	PQ5	PQ6	PQ7	PQ8	PQ9	PQ10	PQ11	RQ1	RQ2	RQ3	RQ4	RQ5	RQ6
**Participant**																	
**Gr3D1**	3	3	3	2	7	3	3	3	3	3	3	2	4	3	5	4	4
**Gr3D2**	4	3	5	5	3	4	3	3	3	4	3	1	4	3	5	4	3
**Gr3D3**	5	4	6	4	4	6	4	5	4	6	4	1	3	4	4	3	4
**Gr3D4**	4	3	3	4	5	4	4	4	4	4	4	2	1	3	5	3	2
**Gr3D5**	3	2	3	3	5	4	3	3	3	4	2	1	2	2	6	3	4
**Gr3D6**	5	4	4	5	3	5	4	5	4	5	4	1	5	2	6	6	5
**Gr3D7**	2	2	1	2	6	3	1	2	1	3	1	1	2	2	6	2	2
**Gr3D8**	4	4	3	4	3	4	3	2	3	4	3	1	4	1	7	4	5
**Gr3D9**	4	3	2	4	5	4	4	3	4	4	4	1	3	2	6	4	3
**Gr3D10**	3	2	2	3	6	3	2	4	2	3	2	1	4	1	7	5	5
**Gr3D11**	6	5	6	7	2	5	5	5	5	5	5	1	2	2	6	3	4
**Gr3D12**	4	3	4	6	3	5	3	4	3	5	3	1	3	3	5	3	4
**Gr3D13**	5	4	3	5	2	6	4	5	3	6	4	1	3	2	6	4	4
**Gr3D14**	3	2	2	3	4	4	3	3	3	4	3	1	3	2	6	3	3
**Gr3D15**	3	3	2	3	5	4	2	4	2	4	2	3	4	3	5	4	5
**Gr3D16**	3	2	2	3	6	4	3	4	2	4	3	1	5	2	6	5	5
**Gr3D17**	6	5	4	4	2	6	6	6	5	6	6	1	7	1	7	6	5
**Gr3D18**	4	4	1	3	5	4	3	4	2	4	3	1	4	4	4	4	4
**Gr3D19**	3	3	4	2	6	4	2	4	1	4	2	1	4	1	7	5	5
**Gr3D20**	1	1	1	1	6	3	1	2	1	3	1	1	6	2	6	5	5
**Gr3D21**	5	4	4	4	3	6	6	5	5	6	6	1	1	3	5	2	2
**Gr3D22**	3	2	1	3	3	3	2	3	2	3	2	1	5	3	5	5	4
**Gr3D23**	3	2	1	4	6	2	2	1	2	2	2	1	4	1	7	4	4
**Gr3D24**	4	3	3	4	4	5	4	4	3	5	4	1	6	2	6	6	5
**Gr3D25**	2	1	1	1	5	2	3	2	3	2	3	2	3	3	5	4	4
**Gr3D26**	5	4	4	5	2	6	5	5	4	6	4	1	3	3	5	3	2
**Gr3D27**	4	4	2	4	3	5	5	5	4	5	5	1	5	1	7	5	6
**Gr3D28**	5	4	4	5	4	5	5	4	5	5	5	1	1	4	4	2	2
**Gr3D29**	5	5	4	4	5	5	4	5	4	5	4	1	1	4	4	2	3
**Gr3D30**	5	5	3	3	3	5	4	4	3	5	4	1	2	2	6	3	3
**Gr3D31**	3	2	2	3	5	4	4	3	3	4	4	1	3	3	5	3	4
**Gr3D32**	4	4	4	4	5	5	4	6	4	5	4	1	4	1	7	5	5
**Gr3D33**	2	1	3	1	4	2	2	2	1	2	2	1	3	2	6	4	4
**Gr3D34**	3	2	2	2	4	3	2	3	2	3	2	1	5	2	6	5	4
**Gr3D35**	3	3	2	3	5	3	3	3	3	3	2	1	3	1	7	3	4

**Table A3:** Responses to the SA questionnaire and scores of the LT knowledge test of the GrV experimental group.

Questionnaire	LT		SA											
Question	LT1	LT2	SA1	SA2	SA3	SA4	SA5	SA6	SA7	SA8	SA9	SA10	SA11	SA12
**Participant**														
**GrV1**	24	−1	3	2	2	3	2	2	2	1	1	2	5	4
**GrV2**	27	−1	4	3	4	5	3	3	5	3	2	3	4	3
**GrV3**	18	−6	2	2	3	5	2	5	3	1	1	1	3	3
**GrV4**	22	−4	3	2	3	3	3	3	4	1	1	1	3	4
**GrV5**	20	−6	2	1	3	4	4	3	7	1	1	1	2	3
**GrV6**	29	−6	3	3	3	3	1	3	4	1	1	2	3	3
**GrV7**	23	−4	5	3	2	2	4	2	2	1	2	1	4	4
**GrV8**	23	−4	1	2	4	5	5	6	5	1	2	2	2	4
**GrV9**	15	−2	3	2	1	2	1	3	4	1	1	2	3	4
**GrV10**	24	−7	4	3	4	5	2	4	6	1	2	2	4	2
**GrV11**	24	−3	4	4	3	5	3	3	5	2	2	2	4	5
**GrV12**	22	−2	6	4	5	5	3	5	5	1	3	1	4	4
**GrV13**	28	−2	3	2	2	3	1	2	2	1	1	1	5	4
**GrV14**	22	−6	3	2	4	5	4	5	3	1	2	2	3	3
**GrV15**	26	−4	1	1	4	6	5	4	6	1	2	1	2	3
**GrV16**	18	−5	3	2	3	4	4	4	5	1	1	1	4	4
**GrV17**	17	−3	4	3	3	4	3	3	4	1	2	2	4	4
**GrV18**	26	0	2	2	5	6	3	5	6	2	3	4	3	5
**GrV19**	26	−2	5	3	1	2	1	1	1	2	1	2	5	3
**GrV20**	20	−3	1	1	2	3	2	3	4	1	1	1	2	5
**GrV21**	19	−1	3	3	5	6	4	4	6	1	2	2	4	3
**GrV22**	30	−7	3	2	4	4	2	3	5	1	1	1	5	5
**GrV23**	23	−2	4	3	4	5	4	4	6	1	2	2	4	3
**GrV24**	16	−3	5	5	3	4	2	3	3	3	2	4	5	4
**GrV25**	22	−6	3	2	3	4	3	4	6	1	1	1	4	4
**GrV26**	26	-	-	-	-	-	-	-	-	-	-	-	-	-
**GrV27**	25	−4	2	3	6	6	4	6	6	2	3	3	3	4
**GrV28**	23	−1	2	1	2	2	3	3	3	2	1	3	3	5
**GrV29**	20	−4	4	1	3	3	3	3	4	2	2	2	5	4
**GrV30**	17	−4	2	1	3	3	2	2	4	2	1	2	4	5
**GrV31**	20	0	2	1	3	3	1	3	4	2	1	3	2	3
**GrV32**	20	1	2	1	6	6	5	6	6	1	2	1	3	4
**GrV33**	20	−4	6	1	1	1	1	3	2	2	1	3	5	5
**GrV34**	19	−6	1	1	2	2	4	3	1	1	1	2	3	4
**GrV35**	18	−6	4	2	3	3	2	4	3	1	1	1	5	4

The LT1 column shows the number of correct answers given to the LT test in the first round. The LT2 column shows the difference between the number of correct answers given to the LT test in the first round and in the second round three months after the training.

**Table A4:** Responses to the SA questionnaire and scores of the LT knowledge test of the Gr3D experimental group.

Questionnaire	LT		SA											
Question	LT1	LT2	SA1	SA2	SA3	SA4	SA5	SA6	SA7	SA8	SA9	SA10	SA11	SA12
**Participant**														
**Gr3D1**	19	−1	3	3	3	4	1	4	3	2	1	1	5	6
**Gr3D2**	24	−2	3	3	3	5	3	4	5	1	2	1	3	5
**Gr3D3**	25	−3	3	2	5	5	4	4	5	2	2	2	4	4
**Gr3D4**	22	−2	2	1	4	5	5	3	3	3	2	4	2	4
**Gr3D5**	28	−2	2	1	3	5	3	4	4	1	1	2	2	3
**Gr3D6**	21	−3	6	4	4	5	6	5	5	2	3	2	4	5
**Gr3D7**	21	−1	2	1	2	2	2	2	4	2	1	3	2	5
**Gr3D8**	29	−1	3	3	3	4	4	5	3	1	2	1	3	5
**Gr3D9**	25	−2	4	2	4	5	3	4	4	2	2	2	3	4
**Gr3D10**	24	−1	4	3	2	3	3	3	4	1	1	2	4	6
**Gr3D11**	23	−1	2	1	7	6	6	6	7	1	2	2	2	4
**Gr3D12**	22	−2	4	4	4	5	4	5	6	2	2	1	5	4
**Gr3D13**	25	−2	3	3	6	5	6	3	4	1	2	2	4	5
**Gr3D14**	20	−3	3	2	3	4	2	4	4	1	1	1	2	4
**Gr3D15**	20	−1	4	3	4	4	4	3	4	2	2	1	3	5
**Gr3D16**	26	−2	5	4	3	4	2	4	3	3	2	3	4	6
**Gr3D17**	20	−2	7	6	7	7	6	5	5	3	4	4	7	7
**Gr3D18**	24	−2	5	3	3	4	5	3	4	2	2	1	5	5
**Gr3D19**	22	0	4	3	3	4	3	3	4	1	1	2	4	5
**Gr3D20**	23	−3	7	5	2	2	1	3	2	3	2	4	7	6
**Gr3D21**	18	−2	2	1	6	6	5	5	4	2	2	3	2	3
**Gr3D22**	22	−2	5	4	4	4	2	3	3	1	2	2	4	6
**Gr3D23**	27	−2	4	3	2	3	1	5	4	2	1	2	5	4
**Gr3D24**	20	−2	6	5	4	5	3	4	4	3	3	4	6	6
**Gr3D25**	24	−3	4	2	2	3	3	2	3	1	1	2	3	3
**Gr3D26**	22	−3	3	2	5	5	5	4	5	1	2	1	4	6
**Gr3D27**	24	−2	6	4	4	5	3	4	3	2	2	3	6	6
**Gr3D28**	23	−3	3	2	5	5	4	5	5	2	2	3	2	4
**Gr3D29**	25	−3	2	2	5	5	5	5	6	2	2	2	2	5
**Gr3D30**	27	−3	3	1	6	5	5	6	6	1	2	2	4	2
**Gr3D31**	26	−4	3	2	3	3	3	4	4	2	1	2	4	4
**Gr3D32**	27	−3	4	3	5	5	4	5	4	1	2	1	6	6
**Gr3D33**	22	−3	3	2	3	2	3	4	4	1	1	1	4	4
**Gr3D34**	25	−4	4	4	3	3	3	4	4	2	2	2	3	5
**Gr3D35**	23	−3	4	3	3	3	2	3	3	1	1	2	4	4

The LT1 column shows the number of correct answers given to the LT test in the first round. The LT2 column shows the difference between the number of correct answers given to the LT test in the first round and in the second round three months after the training.
